# Supplementary material for: RpoS is a pleiotropic regulator of motility, biofilm formation, exoenzymes, siderophore and prodigiosin production, and trade-off during prolonged stationary phase in Serratia marcescens
Source: PLoS One. 2020 Jun 2;15(6):e0232549. doi: 10.1371/journal.pone.0232549 (PMC7266296; doi:10.1371/journal.pone.0232549)
Supplement: S1 Table — (PDF) [file pone.0232549.s001.pdf]

S1 Table. Primers used in this study.

Primers[a]	Sequence (5′ -3′)	Reference or source
**For *rpoS* deletion**		
F-rpoS-U1	GGAATTCAGGTGGGAATAAGGGTGTC	This study
F-rpoS-D1	AAGCGGAAGCAAATCGTTCTCGCTGGTCTC	This study
F-rpoS-U2	AGAACGATTTGCTTCCGCTTCTCCAC	This study
F-rpoS-D2	CAAGCTTGCGTCAAGCCAATCTCC	This study
F-rpoS-W	CAGCACCGTCAGCAACA	This study
R-rpoS-W	CGTCAAGCCAATCTCCC	This study
F-rpoS-c	GGAATTCCGCGTAGTGTATGCAGGCAAC	This study
R-rpoS-c	CAAGCTTTTATTCGCGGAACAGCGCC	This study
**For RT-qPCR**		
RT-16s-F	CGAACCGCTGGCAACAA	This study
RT-16s-R	ACGCGAAGAACCTTACCTACTCT	This study
RT-pigA-F	ATGGCTTTATGGGCGTGTC	This study
RT-pigA-R	TGAAGGTCAGTTCGCTCCAC	This study
RT-luxS-F	GCTGAAAGTGACCGACCAGC	This study
RT-luxS-R	CGCCGTTATCCAGAATGTGC	This study
RT-fliC-F	CTGGCTACCCTGGACAAAGC	This study
RT-fliC-R	GGATACGGGACTGAGAAGCG	This study
RT-flhD-F	CCGCAATGTTTCGCCTG	This study
RT-flhD-R	GGCGTTCGATGGTCTGGC	This study
RT-ompC-F	AACGGGCTTCACTATTTCTCCA	This study
RT-ompC-R	TTCAGCGTGGTTCAGGTTCG	This study
RT-ompF1-F	ACCTATGTTCGTTTCGGCTTCA	This study
RT-ompF1-R	GTGCCCTGAGATTCGGAGTG	This study
RT-ompF2-F	ACCAGGGCAAGAACGAAAAC	This study
RT-ompF2-R	GGTGCGGTTGGAAGAAGC	This study

[a] F, forward primer; R, reverse primer.
