# Supplementary material for: RpoS is a pleiotropic regulator of motility, biofilm formation, exoenzymes, siderophore and prodigiosin production, and trade-off during prolonged stationary phase in Serratia marcescens
Source: PLoS One. 2020 Jun 2;15(6):e0232549. doi: 10.1371/journal.pone.0232549 (PMC7266296; doi:10.1371/journal.pone.0232549)
Supplement: S2 Table — (PDF) [file pone.0232549.s002.pdf]

S2 Table. General genome features of *S. marcescens* strains 1912768R, 1912768WR and 1912768W.

**Genome feature**	**Value**		
	**1912768R**	**1912768WR**	**1912768W**
Gene number	4927	4998	4997
No. of all scaffolds	1	48	39
Bases in all scaffolds	5117289	5075193	5081421
Gene total length (bp)	4478904	4422783	4450470
Gene average length (bp)	909.05	884.91	890.63
Gene density (kb)	0.96	0.98	0.98
GC content in gene region (%)	61.20	61.25	61.24
Gene/Genome (%)	87.52	87.15	87.58
Intergenetic region length (bp)	638385	652410	630951
GC content in intergenetic region (%)	49.60	49.88	49.53
Intergenetic length/Genome (%)	12.48	12.85	12.42
