# Supplementary figures and images for: RpoS is a pleiotropic regulator of motility, biofilm formation, exoenzymes, siderophore and prodigiosin production, and trade-off during prolonged stationary phase in Serratia marcescens
Source: PLoS One. 2020 Jun 2;15(6):e0232549. doi: 10.1371/journal.pone.0232549 (PMC7266296; doi:10.1371/journal.pone.0232549)

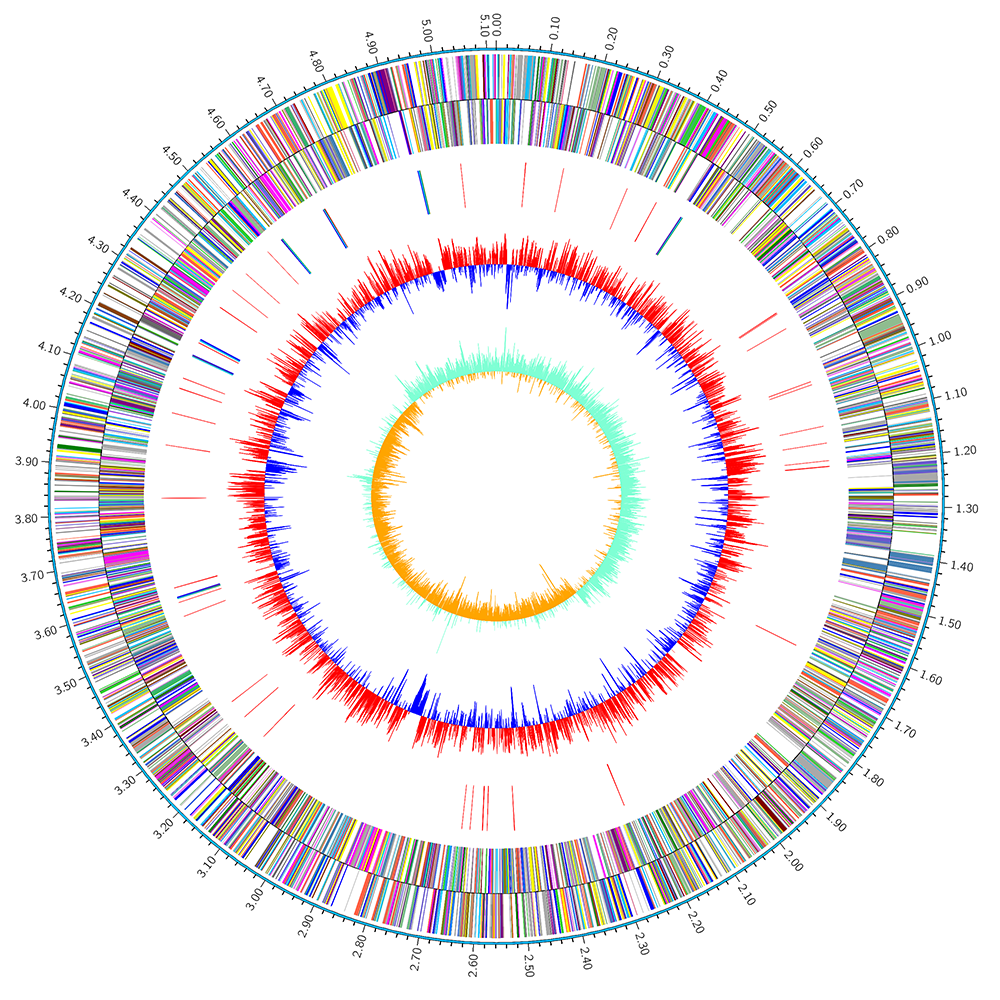

Supplement: S1 Fig — The outermost circle is the genome size, each scale is 0.1 Mb; the second and third circles are the CDS on the positive and negative strands, and different colors indicate the different COG functional classification of the CDS; the fourth circle is rRNA and tRNA; the fifth circle is the GC content. The outward red part indicates that the GC content in this area is higher than the average GC content of the whole genome. The higher the peak, the greater the difference from the average GC content. The inward blue part indicates that the regional GC content is lower than the average GC content of the whole genome; the innermost circle is the GC-skew value. (TIF) [file pone.0232549.s003.tif]

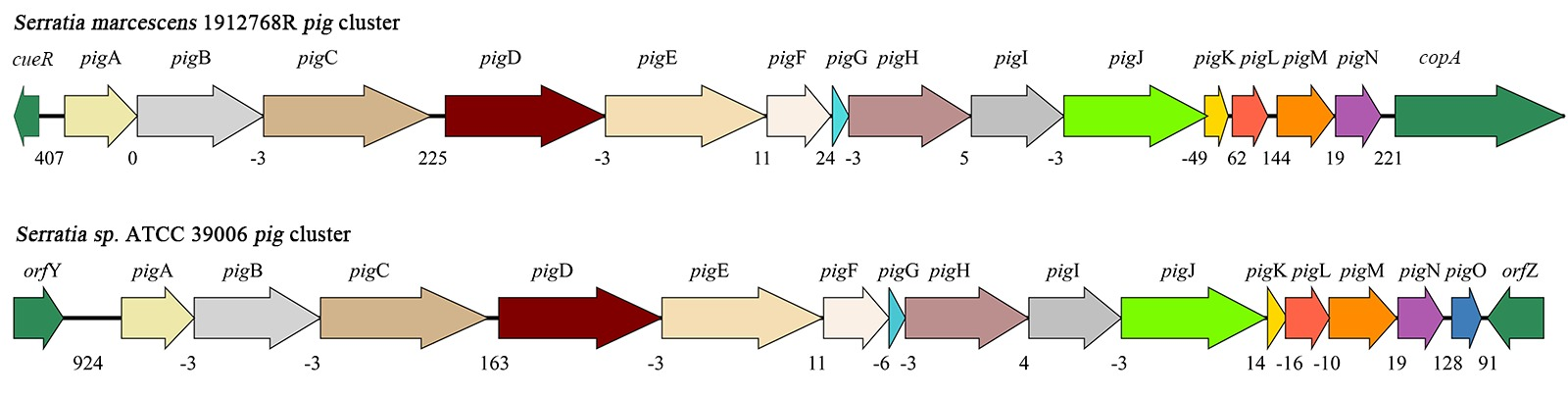

Supplement: S2 Fig — (TIF) [file pone.0232549.s004.tif]

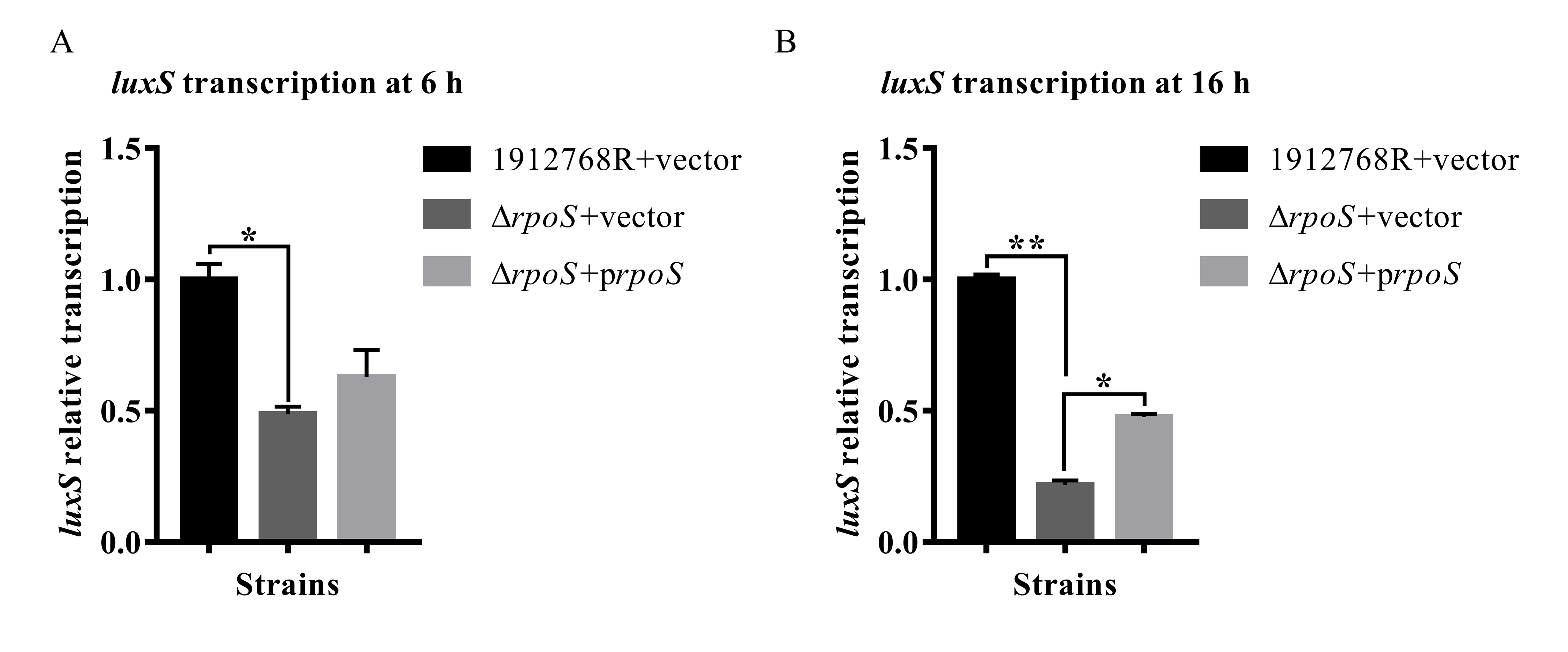

Supplement: S3 Fig — Relative transcription of gene rpoS from WT strain, ΔrpoS and rpoS complemented strain at 6 h (a) and 16 h (b). (TIF) [file pone.0232549.s005.tif]
